# Is socio-demographic status, body mass index, and consumption of food away from home associated with high sodium intake among adults in Malaysia?: findings from the Malaysian Community Salt Survey (MyCoSS)

**DOI:** 10.1186/s41043-021-00236-z

**Published:** 2021-05-31

**Authors:** Ruhaya Salleh, Shubash Shander Ganapathy, Norazizah Ibrahim Wong, Siew Man Cheong, Mohamad Hasnan Ahmad, Lalitha Palaniveloo, Fatimah Othman, Azli Baharudin, Megat Rusydi Megat Radzi, Rusidah Selamat, Nur Shahida Abd. Aziz, Rashidah Ambak, Tahir Aris

**Affiliations:** 1 Center for Nutrition and Epidemiology Research, Institute for Public Health, National Institutes of Health, Ministry of Health Malaysia, Jalan Setia Murni U13/52, Seksyen U13, 41070 Shah Alam, Malaysia; 2 Sector of Biostatistics and Data Repository, National Institutes of Health, Ministry of Health Malaysia, 41070 Shah Alam, Malaysia; 3 Dietetic and Food Service Department, Hospital Sultanah Aminah, 80100 Johor Bahru, Johor Malaysia; 4 Nutrition Division, Ministry of Health Malaysia, Federal Government Administrative Centre, 62590 Putrajaya, Malaysia; 5 Institute for Medical Research, National Institute of Health, No 1, Jalan Setia Murni, U13/52, Seksyen U13, Setia Alam, 40170 Shah Alam, Selangor, Malaysia

**Keywords:** Sodium, Eating away from home, Adults, Food frequency questionnaire, Malaysia

## Abstract

**Background:**

Studies have shown that having away from home meals contributes to high sodium intake among young people and those who lived in urban areas. This study aimed to determine the association between dietary sodium intake, body mass index, and away from home meal consumption behaviour among Malaysian adults.

**Methods:**

MyCoSS was a cross-sectional household survey involving 1440 adults age 18 years and above. This study utilized stratified cluster sampling to obtain a nationally representative sample. Data was collected between October 2017 and March 2018. Socio-demographic information, dietary assessment using food frequency questionnaire (FFQ), and away from home meal consumption were assessed through a face-to-face interview by trained health personnel. Descriptive analysis and logistic regression were applied to identify the association of socioeconomic status and away from home meal consumption with dietary sodium intake.

**Results:**

A total of 1032 participants completed the FFQ, with a mean age of 48.8 + 15.6 years. Based on the FFQ, slightly over half of the participants (52.1%) had high sodium intake. Results showed that 43.6% of participants consumed at least one to two away from home meals per day, while 20.8% of them had their three main meals away from home. Participants aged less than 30 years old were the strongest predictor to consume more sodium (adjusted OR: 3.83; 95%CI: 2.23, 6.58) while those of Indian ethnicity had significantly lower sodium intake. Surprisingly, having three away from home meals per day was not associated with high dietary sodium intake, although a significant association (crude OR; 1.67, 95% CI: 1.19, 2.35) was found in the simple logistic regression. Obese participants were less likely to have high dietary sodium intake compared with the normal BMI participants in the final model.

**Conclusion:**

Over half of the participants consumed sodium more than the recommended daily intake, especially those who consumed three away from home meals. However, there was no significant association between high sodium intake and having three away from home meals per day. The promotion of healthy cooking methods among the public must continue to be emphasized to reduce the dietary sodium intake among Malaysian adults.

## Background

Modern lifestyle and time scarcity have contributed to an increasingly prevalent habit of eating out in Malaysia, especially in the urban areas as indicated by the rise in food expenditure from 10.7 to 11.3% between 2016 and 2019 across all household income classes [1]. Eating meals away from home was associated with a relatively higher intake of energy, sodium, and fat and a lower intake of micronutrients. As the frequency of eating meals away from home increases, the risk of non-communicable diseases such as hypertension and obesity also increases [2–4]. It is well-known that one of the preventable risk factors for high blood pressure is excessive sodium intake from the diet [5, 6]. Based on the National Health and Morbidity Survey conducted in 2019, the prevalence of adults having hypertension was 30.0% [7].

A local study conducted by the Institute for Public Health (IPH) in 2014 found that the median dietary intake of sodium was 1935 mg/day among Malaysian working adults [8]. Men were found to consume a slightly higher amount of sodium (1970 mg) compared to women (1914 mg). IPH conducted a subsequent survey in 2015 [9], where the sodium intake was assessed based on 24-h urinary excretion among the staff of the Ministry of Health Malaysia and had found that the sodium intake was 1.5 fold higher (3446 mg/day) than the previous finding. This difference may be because sodium captured in the 2014 study was based on dietary intake, while the study conducted in 2015 used urinary sodium. This study identified that the three main high sodium-containing foods commonly consumed by the working adults are light soy sauce (225.3 mg/day), fried rice (162.8 mg/day), and an omelet (156 mg/day). The finding by IPH (2015) was alarming because the consumption of sodium exceeded the amount recommended by the World Health Organization (WHO) (<2000 mg/day) and the Recommended Nutrient Intake (RNI) for Malaysia (2300 mg/day) [10]. Another narrative review study among six southern countries, including Malaysia, revealed that sodium intake assessed using 24-h diet recall among Malaysian adults was 2575 mg/day, with slightly higher consumption among males compared to their female counterparts [11].

Several studies have been conducted to investigate the relationship between meal patterns and sodium intake. An Australian study showed that dinner contributed to the highest dietary sodium intake per day compared to lunch or breakfast [12]. Shanghai Diet and Health Survey study among adults aged 18 years and above reported that eating at restaurants was associated with higher sodium intake [13]. Another study at Minneapolis/St. Paul metropolitan area of Minnesota, by Larson (2011), reported those consumed foods prepared away from home to tend to have higher sodium intake sodium [14]. Although there were studies from overseas to explore the link between having away from the home meals and sodium intake as well as blood pressure, there is a lack of local study to investigate the association between having away from the home meal and dietary sodium intake among Malaysian adults. Therefore, the objectives of the present study were to examine the associations between socio-demographic status, body mass index, and away from home meal consumption with high sodium intake in a nationally representative sample of Malaysian adults.

## Methods

Data from the Malaysian Community Salt Survey (MyCoSS), secondary data which was collected between October 2017 and March 2018 was utilized. MyCoSS was a cross-sectional, nationally representative household survey involving 1440 adults aged 18 year and above. A stratified cluster sampling method was applied to ensure national representativeness by strata. The population included residents from non-institutional living quarters (LQs), while institutionalized residents such as occupants of hotels, hostels, hospitals, and prisons were excluded from the survey. Participants who were pregnant, menstruating during the urine collection period, diagnosed with kidney failure, heart failure, or liver disease, as well as those who had difficulty with urine collection and had recently taken diuretics within the 4 weeks preceding the study were also excluded from the study.

The sample size was calculated using the formula for estimating population prevalence. Findings from the previous study about salt intake among health workers in Malaysia (MySalt 2015) were used for sample size calculation for this study [9]. Analyses were done based on all objectives, and the largest sample size was the objective of knowledge about the impact of high salt intake on health problems which was 60%. The optimum sample size for a stratum was 520 with a confidence level of 95% and an estimated design effect of 1.5. With consideration of 25% non-response rate, the sample size was inflated to 650 for one stratum. Therefore, a total of 1300 participants were needed to represent the adult’s residents in rural and urban in Malaysia.

### Socio-demographic characteristics, body mass index, and away from home meals

Socio-demographic information collected in the survey included gender, ethnicity, marital status, locality, education levels, employment status, and household income.

Trained health personnel measured the body weight and height of each participant in light clothing without shoes using a calibrated digital weighing scale (Tanita HD 319, Japan) and stadiometer (SECA 213, Germany). Body mass index (BMI) was calculated and categorized based on the WHO 1998 Classification [15].

Meal pattern was assessed through interviews using a pre-tested questionnaire developed for this study. Meal pattern refers to the commonly practised meal by the respondents. Data was collected on main meal intake (breakfast, lunch, and dinner) in a day and sources of those meals (at home or away from home). Having breakfast, lunch, and dinner away from home were then grouped into the category of “having three away from home meals.”

### Dietary sodium intake assessment using Food Frequency Questionnaire (FFQ)

A pre-tested FFQ with 104 high sodium content food items (≥0.25 g salt/serving or ≥0.1 g sodium/serving) was used to determine the dietary sodium intake. The FFQ was developed based on food intake from 24-h diet recall assessment from a previous study in Malaysia [9]. This FFQ contains 11 food groups, which are meat and products; fish/seafood and products; eggs; spread; local delicacies bread; snacks; seasoning/flavoring/sauces; fast food; cooked food; other cooked foods and canned foods. Participants reported their frequency intake (daily or weekly or monthly) and portion consumed for each food item in the FFQ. A food album was developed to assist the participants in identifying food items in the FFQ. Most of the food items consumed by the respondents can be analyzed using Malaysia nutritionist pro database. If the food was not captured in the database, other references such as the Malaysia Food Composition Database (MyFCD) and Singapore Food Database were used. Household measurement utensils (such as cups, plates, spoons, and bowl) were used to estimate the portion size of the food intake. Dietary sodium intake was calculated using the formula below [16]:

(Frequency of food item intake /day) × (portion size in gram) × (sodium content/100 g)/100).

For this study, high dietary sodium intake defined as and categorized as ≥2000 mg/day.

### Statistical analysis

All statistical analysis was performed using the IBM SPSS version 23 (IBM Corp., Armonk, NY, USA). Descriptive statistics were computed, and data was tabulated according to socio-demographic status and related variables. Logistic regression analysis was conducted to identify the association between high sodium intake and socio-demographic characteristics, BMI, and away-from-home meal consumption. Socio-demographic characteristics, away from home meals and BMI, were the independent variables and dietary sodium intake derived from FFQ was the dependent variable in this study. All statistical analysis was described using a 95% confidence interval, and *p* value < 0.05 was considered to be significant.

## Results

### Socio-demographic characteristics, away from home meal consumption, and sodium intake of the participants

Of 1440 participants, 1032 participants completed and answered their food intake through Food Frequency Questionnaires (FFQ). Table 1 depicts the socio-demographic characteristics, away from home meal consumption, and dietary sodium intake of the participants. Majority of the participants were aged 50 years and above (52.1%) and were females (59.1%). Almost two-thirds of the participants were of Malay (63.0%) ethnicity, and most of them were married (72.5%). The participants from rural areas were slightly more (58.5%) than from urban areas. There were higher percentages of participants who completed secondary school (47.8%), earned household income less than RM 1000 per month (31.2%), and were housewives (28.5%). More than half of the participants were found to be overweight or obese (61.0%). Having breakfast away from home (55.0%) was the most common meal away from home while lunch and dinner only represent 35.6% and 34.4%, respectively. Besides, 64.4% of participants consumed at least one meal away from home, while 20.8% of participants reported consuming all three meals away from home. Overall, 52.1% of participants were found to have high dietary sodium intake.

**Table 1:** Socio-demographic characteristics, away from home meal consumption, and sodium intake of the participants (*n*=1032)

	Frequency (n)	%	Sodium intake		
			Mean	95% CI	
				Lower	Upper
**Age group** (mean ± SD = 48.8 ± 15.63) years					
Less than 30	143	13.9	3429.7	3024.14	3835.19
30 to <40	176	17.1	3144.2	2847.01	3441.49
40 to <50	175	17.0	2625.5	2159.69	3091.35
50 and above	538	52.1	2277.6	2042.05	2513.17
**Gender**					
Male	422	40.9	2889.3	2633.89	3144.73
Female	610	59.1	2402.0	2193.95	2610.11
**Ethnicity**					
Malay	650	63.0	2694.7	2467.21	2922.30
Chinese	114	11.0	2720.3	2381.89	3058.69
Indian	63	6.1	1654.1	1385.19	1923.09
Bumiputera Sabah	110	10.7	3346.5	2948.66	3744.32
Bumiputera Sarawak	77	7.5	2521.9	1938.24	3105.64
Others	18	1.7	2412.5	1596.24	3228.68
**Marital Status**					
Never married	132	12.8	3189.6	2722.82	3656.40
Married	747	72.5	2645.6	2444.57	2846.68
Separated / Widower	152	14.7	2044.0	1688.12	2400.03
**Strata**					
Urban	428	41.5	2686.4	2468.41	2904.42
Rural	604	58.5	2505.6	2323.37	2687.85
**Education Level**					
None	96	9.3	2177.7	1569.94	2785.43
Primary	215	20.8	2113.3	1874.19	2352.47
Secondary	493	47.8	2780.7	2509.80	3051.50
Tertiary	228	22.1	2872.2	2569.82	3174.65
**Occupation**					
Public	143	13.9	2622.1	2254.63	2989.56
Private	168	16.3	3251.7	2756.76	3746.56
Self-employed	228	22.1	2729.6	2464.07	2995.08
Housewives	294	28.5	2369.4	2099.08	2639.74
Others (Unemployed/students)	199	13.7	2364.1	2014.68	2713.47
**Household income (RM/month)**					
<RM 1000	322	31.2	2341.4	2003.03	2679.68
RM 1000-1999	198	19.2	2369.4	2075.32	2663.52
RM 2000-2999	169	16.4	2674.1	2322.27	3025.90
RM 3000-3999	124	12.0	2880.7	2303.39	3458.09
>RM 4000	219	21.2	2964.5	2660.41	3268.63
**Body Mass Index (kg/m2)**					
Underweight	45	4.4	3022.7	2419.59	3625.80
Normal	357	34.6	2829.6	2489.05	3170.11
Overweight	376	36.4	2587.1	2303.39	2870.88
Obese	254	24.6	2401.1	2116.58	2685.65
**Away from home meal consumption**					
Having breakfast away from home					
Yes	568	55.0	2708.4	2451.33	2965.42
No	464	45.0	2450.9	2184.31	2717.40
Having lunch away from home					
Yes	367	35.6	2889.9	2612.11	3167.70
No	665	64.4	2354.5	2138.39	2570.60
Having dinner away from home					
Yes	280	27.1	3052.1	2745.39	3358.82
No	545	66.1	2487.1	2278.45	2695.70
**Number of away from home meal**					
Not at all	367	35.6	2421.9	2166.86	2677.02
One to two meals	450	43.6	2485.4	2237.66	2733.03
Three meals	215	20.8	3145.7	2778.73	3512.64
**Total sodium intake (mg/day)**					
(mean ± SD = 2608.6 ± 2056.3)					
<2000mg	494	47.9	-	-	-
>2000mg	538	52.1	-	-	-

Table 2 shows that younger age adults were more likely to have high dietary sodium intake (<30 years old: aOR = 3.83, *p*<0.001, 30 to <40 years old: aOR= 2.75, *p*<0.001, and 40 to <50 years old, aOR = 1.67, *p*=0.009) compared to the adults aged 50 years and above. There was an association between sodium intake and gender, whereby males had a 32% higher tendency to consume more sodium daily compared to female (crude OR = 1.32, *p* value = 0.030). However, this association was not seen in the multivariate model. Those of Indian ethnicity consumed significantly lower dietary sodium compared to those of Malay ethnicity. The results indicate that participants of the Indian ethnicity had a 54% lower tendency to consume high sodium foods compared to Malays (adjusted OR = 0.46, *p* value = 0.009). The association between sodium intake and marital status was also found in the present study whereby, participants who were separated or widowed had a 62% lower tendency to consume high sodium foods compared to single participants (crude OR = 0.38, *p* value < 0.001). However, the associations were not significant in the final model. Surprisingly, the present study also found that participants with secondary and tertiary education had a higher chance to over-consume sodium in a day. Participants with tertiary education had 2.43 times higher tendency to over-consume sodium compared to those with no formal education (crude OR = 2.43, *p* value < 0.001).

**Table 2:** Associated factors of high sodium intake by socio-demographic characteristics and away from home meal consumption

Variable	Category	Crude OR	95% CI		***P*** value	Adjusted OR	95% CI		***P*** value
			Lower	Upper			Lower	Upper	
**Age (years)**	Less than 30	3.50	2.36	5.25	**0.001**	3.83	2.23	6.58	**<0.001**
	30 to <40	2.87	2.01	4.11	**0.001**	2.75	1.82	4.16	**<0.001**
	40 to <50	1.68	1.19	2.37	**0.003**	1.67	1.14	2.56	**0.009**
	50 and above	1.00				1.00			
**Gender**	Male	1.32	1.03	1.69	**0.030**	1.27	0.92	1.76	0.145
	Female	1.00				1.00			
**Ethnicity**	Malay	1.00				1.00			
	Chinese	1.17	0.78	1.74	0.450	1.21	0.78	1.87	0.388
	Indian	0.46	0.26	0.79	**0.005**	0.46	0.25	0.82	**0.009**
	Others	1.12	0.82	1.54	0.482	0.99	0.70	1.41	0.976
**Marital status**	Never married	1.00				1.00			
	Married	0.70	0.48	1.02	0.061	1.63	0.99	2.69	0.056
	Separated/widow/widower	0.38	0.23	0.61	**<0.001**	1.26	0.68	2.34	0.455
**Strata**	Urban	1.12	0.87	1.43	0.385	1.09	0.82	1.44	0.569
	Rural	1.00				1.00			
**Education level**	No formal education	1.00				1.00			
	Primary education	1.19	0.73	1.94	0.495	1.04	0.62	1.74	0.886
	Secondary education	1.79	1.15	2.80	**0.011**	1.17	0..72	1.91	0.533
	Tertiary education	2.43	1.50	3.96	**<0.001**	1.19	0.66	2.15	0.564
**Occupation**	Public sector	1.00				1.00			
	Private sector	1.21	0.77	1.91	0.414	1.28	0.76	2.15	0.353
	Self employed	0.86	0.56	1.31	0.470	1.11	0.69	1.81	0.666
	Housewives	0.65	0.43	0.97	**0.036**	1.15	0.69	1.91	0.592
	Others	0.65	0.42	1.01	0.053	0.99	0.59	1.65	0.953
**Body Mass Index (BMI)**	Underweight	0.98	0.52	1.84	0.950	0.76	0.38	1.50	0.429
	Overweight	0.72	0.54	0.97	**0.030**	0.75	0.55	1.02	0.069
	Obese	0.59	0.43	0.82	**0.002**	0.60	0.43	0.86	**0.005**
	Normal	1.00				1.00			
**Household income**	<RM 1000	1.00				1.00			
	RM 1000 -1999	1.03	0.72	1.47	0.857	0.76	0.52	1.12	0.167
	RM 2000 - 2999	1.53	1.05	2.22	**0.027**	0.98	0.64	1.48	0.899
	RM 3000 -3999	1.95	1.28	2.98	**0.002**	1.22	0.76	1.96	0.416
	>RM 4000	1.64	1.16	2.32	**0.005**	0.98	0.63	1.52	0.897
**Number of away from home meal**	Not at all	1.00				1.00			
	One to two meals	1.07	0.81	1,41	0.635	1.04	0.76	1.42	0.814
	Three meals	1.67	1.19	2.35	**0.003**	1.29	0.86	1.93	0.222

*OR* odd ratio, *CI* confidence interval, Nagelkerke *R*^2^ = 0.125, Hosmer and Lemeshow Test (*X*^2^ = 4.010, df = 8, *p* = 0.856)

Furthermore, housewives were found to have a 35% lower risk of over-consume sodium compared to those working in the public sector. There was a significant association between sodium intake and household income as those earning RM 2000 and above had a higher tendency to over-consume sodium. Those who earned RM 3000 to RM 4000 per month had 1.95 times higher risk to consume high sodium per day compared to those earning less than RM 1000 per month (crude OR = 1.95, *p* value = 0.002). However, the association was not found in the multivariate model.

By BMI, overweight and obese participants consumed significantly lower dietary sodium. In the multivariate model, obese participants had approximately 40% lower tendency to over-consume sodium in a day compared to participants with normal BMI (adjusted OR = 0.60, *p* value = 0.005). In the multivariate model, having away from home meals, regardless of frequency per day, was not associated with high dietary sodium intake.

## Discussion

According to WHO guidelines on dietary salt intake, adults should consume less than 2000 mg of sodium or 5 g of salt per day [17]. Our present study found that 52.1% of Malaysian adults exceeded this dietary sodium intake recommendation. This problem is alarming as high dietary sodium intake is associated with various non-communicable diseases such as cardiovascular diseases and cancer. Similarly, the National Nutrition Survey from Singapore revealed that more than 60% of adults consumed salt higher than the recommended amount [18]. In contrast, the survey among American adults showed that 89% exceeded the recommendation for sodium intake [19]. Furthermore, excess sodium intake was also reported among the population in Mexico (44%), France (89.1%), and the UK 83.4% [20].

The current study found that adults aged less than 30 years old were three times more likely to consume sodium compared to adults aged 50 and older. This finding was comparable with a study from Malawi, where younger people aged less than 30 years old consumed higher levels of sodium and ate out more often compared to the older population [21]. A study among adults in Minnesota reported that 95% of young adults reported a preference for foods high in sodium, such as burgers and fries, from fast-food restaurants [14].

Those of Indian ethnicity in this study consumed significantly lower sodium compared to Malays. A systematic review of eight observational studies in Malaysia showed that Malay adolescents had a lower diet quality. There is a high chance that this bad dietary practice among adolescents could have been brought into adulthood [22]. Findings from a study conducted among adolescents in Kuantan also reported that more than half of the Malays adolescent skipped meals compared to Chinese participants who never skipped any meals [23]. Malay adolescents had been shown to have a significantly higher preference for Western and local food, while Chinese adolescents showed a higher priority for healthier food [24].

The current study also found that men, in general, tend to have a higher intake of sodium than women, which is most probably because women are more health-conscious and pay more attention to how their everyday life affect their health than men [25]. A study was done among university adults also found that more men (84%) than women (58%) reported typically eating fast food for lunch at least once weekly [26].

Those with higher education and higher household income were found to have high dietary sodium consumption per day. A systematic review supported this finding reporting that only 3% of available literature found that people with higher SES are associated with higher sodium intake [27]. This association has been postulated to be due to lack of time for cooking, leading to increased consumption of outside food, which is high in salt. Young adults or children from higher socioeconomic strata also have more disposable pocket money [28], which leads to frequent eating out. A study in Malaysia also reported that apart from increased opportunities to eat out, the cost of eating out in Malaysia is sometimes lower than the cost of a homemade meal, thus indirectly promoting the likelihood of eating out [29].

The current study also found that overweight and obese participants were significantly less likely to have high dietary sodium intake compared to those with normal BMI. This finding is not parallel with the current literature [30], and the reason underlying these is unclear. Perhaps, it could be due to the influence of other confounders that are not studied in this research. We do not exclude the possibility of underreporting of dietary intake, as this phenomenon has been shown to occur among obese study participants [31]. It also may be due to the bias of dietary sodium intake assessment using FFQ but not 24-h dietary recall or 24-h urinary sodium excretion.

At present, away from home meals are popular in Malaysia and other countries. The present study found that 64.4% of the participants had at least one away from home meal per day, and these findings are similar to the Malaysian Food Barometer Survey [32]. Internationally, due to socioeconomic reasons, there are wide variations in this figure, with a study from Singapore reporting that 77.3% of their citizens eating outside [33]. In comparison, a study in Brazil reported only 24% of people to have at least one away from home meal [34]. In this study, we found no association between away from home meals and dietary sodium intake, which was an exciting finding in our study. It shows that Malaysian use excessive salt during food preparation, even for meals cooked at home. The food choices at home among Malaysians also tend to include food items that are high in salt content. So our findings are essential to both healthcare professionals and policymakers to strategize our programs in accordance with this finding. Dietary advice and health promotion on salt reduction should be tailored not only for patients but should also include food operators and specifically among those who prepare the food at home.

We keep in mind the limitations of this study that the findings are based on FFQ and that certain food items with lower sodium content may be consumed in higher amounts or frequency, but was not included in our FFQ, leading to the inaccuracies of our study findings. We utilized the Nutritionist Pro software available in the Institute for Public Health to determine the sodium content for this analysis. To our knowledge, this is the complete database available in Malaysia for nutritional analysis. The findings are also dependent on recall, as is an inherent limitation of any study of such design. However, to our best knowledge, this study does represent the first nationwide study to assess away from home meals with sodium intake among Malaysian adults.

## Conclusions

More than 50% of Malaysian adults were found to exceed the recommended dietary sodium intake and consumed at least one away from home meal. However, having away from home meal is not significantly associated with high dietary sodium intake. These interesting study findings support the need to explore further the impact of meals and snacks, as well as cooking methods, with dietary sodium. Sodium reduction strategy should focus on all individuals, including those who eat out frequently and individuals who prepare food at home. Moreover, education on dietary sodium reduction should be emphasized among different ethnicities and people of a younger age to prevent the early onset of high blood pressure and other related chronic diseases.

## Data Availability

The datasets used and analyzed during the current study are available from the corresponding author on reasonable requests.

## Abbreviations

MyCoSS: Malaysian Community Salt Survey
BMI: Body mass index
BP: Blood pressure
CVD: Cardiovascular disease
FFQ: Food Frequency Questionnaire
IPH: Institute for Public Health
LQ: Living quarters
RM: Ringgit Malaysia
WHO: World Health Organization
SES: Socioeconomic Status.
